# Gastrointestinal Tract Perineuriomas and Benign Fibroblastic Polyps: Case Report and Comprehensive Systematic Review

**DOI:** 10.1155/crgm/1636142

**Published:** 2025-03-09

**Authors:** Ahmad F. Alenezi, Haitham Jahrami, Yonca Kanber, Talat Bessissow

**Affiliations:** ^1^Department of Medicine, McGill University, 3605 Rue de La Montagne, Montreal H3G 2M1, Quebec, Canada; ^2^Department of Government Hospitals, Ministry of Health, Road 2909, Salmanyia, Manama, Bahrain; ^3^Department of Pathology, McGill University Health Centre, 1001 Boul. Décarie, Rm E04.4130, Montreal H4A3J1, Quebec, Canada; ^4^Division of Gastroenterology, McGill University Health Centre, 1650 Avenue Cedar, #C7-200, Montreal H3G 1A4, Quebec, Canada

**Keywords:** benign fibroblastic polyps, case report, case series, gastrointestinal tract, perineurioma, systematic review

## Abstract

**Background:** Perineurioma is a rare benign peripheral nerve sheath tumor that can arise in various body locations. In the gastrointestinal (GI) tract, perineuriomas are uncommon and have only been reported in case reports and case series. In addition, a new classification suggests reclassifying benign fibroblastic polyps as perineurioma when they show positive markers of perineurial differentiation.

**Objective:** This study aims to enhance understanding of GI tract perineuriomas by presenting a new case and conducting a systematic literature review.

**Methods:** We described a new case of colonic perineurioma and systematically reviewed all case reports and case series on GI perineuriomas and benign fibroblastic polyps with perineurial markers. We searched ScienceDirect, PubMed/MEDLINE, and Web of Science up to May 2024.

**Results:** A total of 148 cases were analyzed, and most of the cases were published in the last decade (2014–2024). The majority were females (59.46%), with a mean age of 51 years (standard deviation [SD] ±14.87). Most GI perineuriomas (87.5%) were in the distal colon, predominantly in the sigmoid/rectosigmoid (56%) and rectum (14%). Outside the colon, the stomach was the most affected site (7 of 10 cases), with fewer cases in the small intestine and esophagus. The two most commonly performed stains were for epithelial membrane antigen (EMA) and glucose transporter 1 (GLUT-1), at 75% and 56% of cases, respectively. Noncolonic perineuriomas were generally larger and more symptomatic than colonic ones. Submucosal polyps were more likely symptomatic than mucosal polyps.

**Conclusion:** Perineurioma in the GI tract is a rare benign polyp mainly identified in the distal colon. Its rarity and limited follow-up data restrict our understanding of recurrence rates. We recommend reporting uncommon polyp locations, detailing polyp morphologies, and using at least two markers for classification.

## 1. Introduction

Perineurioma is a benign peripheral nerve sheath tumor (NST) that arises from perineurial cells, and it was first described in 1978 by Dr. Lazarus [1–3]. These lesions can occur in various locations throughout the human body, including soft tissues and skin, and less frequently in the gastrointestinal (GI) tract [2]. Perineurial cells, which form the protective sheath around peripheral nerves, contribute to the unique histological and immunohistochemical (IHC) profiles that distinguish perineuriomas from other types of neoplasms [4].

Histopathological examination and IHC staining are essential for the accurate diagnosis and differentiation of perineuriomas from other NSTs originating from Schwann cells and fibroblasts [5]. Histologically, perineuriomas are identified as intramucosal proliferations of bland spindle cells with an eosinophilic cytoplasm, oval nuclei, and indistinct cell borders. The entrapment of crypts and associated hyperplastic epithelium are frequently observed along with the absence of nuclear pleomorphism, mitotic activity, and necrosis [6]. IHC staining complements microscopic examination by confirming the presence of specific perineurial markers. Positivity for markers such as epithelial membrane antigen (EMA) [4, 5, 7], collagen IV [7], claudin-1 [4, 7], and glucose transporter 1 (GLUT-1) [7] is commonly used to confirm the diagnosis of perineuriomas.

EMA was initially considered a specific marker for epithelial cells. However, its expression has also been detected in several other cell types, including perineurial cells [8]. Collagen IV, a major component of the basement membrane, provides structural support and separates different tissue layers. Within the context of perineurial cells, the basement membrane is crucial for maintaining the integrity and function of the nerve sheath [9]. Claudin-1, a protein associated with tight junctions, is expressed by perineurial cells and has been reported to be a highly sensitive and specific marker for perineurium and soft tissue perineuriomas [10]. GLUT-1 is another relatively specific marker for perineurial differentiation, as it is found in soft tissue perineuriomas but not in lesions containing fibroblasts, neurons, or smooth muscle cells [10]. These markers are crucial for distinguishing perineuriomas from other histologically similar NSTs, ensuring precise and reliable identification.

In 2004, Eslami-Varzaneh et al. [11] reported 14 cases of new colonic polyps with histologic features indicative of fibroblastic differentiation of spindle cells. They classified these polyps as a new type of mucosal colonic lesion, termed benign fibroblastic polyps. Later, in 2005, Hornick and Fletcher [12] described eight cases of mucosal perineurioma with morphologic features similar to fibroblastic polyps. This study was followed by two more investigations in 2006 and 2008 that were conducted by Groisman and Polak-Charcon [7, 13]. After including a total of 38 patients from both studies, they suggested reclassifying patients previously diagnosed with benign fibroblastic polyps as having extraneural perineurioma and recommended the use of two specific markers of perineurial cells.

In the GI tract, perineuriomas are exceedingly uncommon, and their presentation can be easily overlooked or misdiagnosed due to their subtle clinical manifestations and morphological similarities to other GI polyps/lesions [11, 14, 15]. These polyps are often detected incidentally during routine screening colonoscopy [16]. The majority of cases have been identified in the distal colon (left-sided colon) [16, 17]; however, recent reports have identified the same lesion in different locations within the GI tract, such as the esophagus [18], stomach [19], and small intestine [12, 20].

This study presents a case of colonic perineurioma in the descending colon and reports a systematic review consolidated with a descriptive and subgroup analysis of the included cases and case series of perineurioma and benign fibroblastic polyps in the GI tract. By analyzing the relevant literature on perineuriomas and benign fibroblastic polyps that fit the criteria for perineuriomas, this study aims to provide critical information regarding such infrequent lesions. Therefore, these findings will help pathologists and gastroenterologists better understand the clinical and histopathological aspects of perineuriomas in the GI tract.

## 2. Materials and Methods

### 2.1. Case Report

The current case report was conducted in line with the CAse REport (CARE) guidelines [21] to offer a comprehensive and standardized analysis of the selected clinical case. Informed consent was obtained from the patient to publish the case report and any related images.

### 2.2. Systematic Review

#### 2.2.1. Database Searches

A literature review was conducted between January 2004 and May 2024 following the Preferred Reporting Items for Systematic Reviews and Meta-Analyses (PRISMA) and PRISMA Protocol (PRISMA-P) guidelines [22] using three database websites: ScienceDirect, PubMed/MEDLINE, and Web of Science. The search strategy employed a combination of key terms and phrases, targeting those in List A (e.g., “gastroenterology perineurioma,” “esophageal perineurioma,” “gastric perineurioma,” “small intestine perineurioma,” “colonic perineurioma,” “gastroenterology benign fibroblastic polyp,” “esophageal benign fibroblastic polyp,” “gastric benign fibroblastic polyp,” “small intestine benign fibroblastic polyp,” or “colonic benign fibroblastic polyp”) in conjunction with terms in List B (“case report” or “case series”) for full-text retrieval.

#### 2.2.2. Study Selection

Papers were selected on the basis of the following criteria: publication in a peer-reviewed journal, publication language in English, and histopathological features of perineurioma or benign fibroblastic polyp along with immunohistochemistry positivity for at least one of the following markers: EMA, collagen IV, claudin-1, and GLUT-1.

#### 2.2.3. Data Extraction

The extracted data included the lead author's name, article title, date of publication, age or average age, sex, number of polyps identified, morphology, size of the polyps, depth of the polyps, immunohistochemistry stain positivity, and indication for esophagogastroduodenoscopy (EGD), colonoscopy, or surgical excision.

#### 2.2.4. Quality Assessment

The critical appraisal checklists developed by the Joanna Briggs Institute (JBI) [23] were used in this study to evaluate the quality of the selected case reports and case series. The checklists offered a systematic framework to assess the comprehensiveness of the reports as well as the methodological approach used.

#### 2.2.5. Data Synthesis and Analysis

The descriptive statistics used in this report helped to summarize the collected data. Dichotomous variables were analyzed as frequencies and percentages. Data analysis was thus performed using the Statistical Package for Social Sciences (SPSS) Version 28.0 [24]. *T* tests, chi-square tests, and *p* values were used for comparisons between groups in the subgroup analysis. Binary variables including anatomical location, IHC staining (EMA, GLUT-1, collagen IV, and claudin-1), and clinical presentation (symptomatic vs. asymptomatic) were analyzed. Statistical analyses included phi coefficient calculations for categorical associations and contingency tables. Location data were categorized into proximal and distal colon involvement, with polyp size recorded in millimeters.

## 3. Results

### 3.1. Case Report

A 68-year-old female with no significant past medical history presented to the gastroenterology outpatient clinic after being referred by her family physician following a positive fecal occult blood test. The physical examination was unremarkable, and she reported no history of abdominal pain or rectal bleeding. Complete blood counts, liver function tests, coagulation profiles, and electrolyte levels were within normal limits.

**Figure 1:**
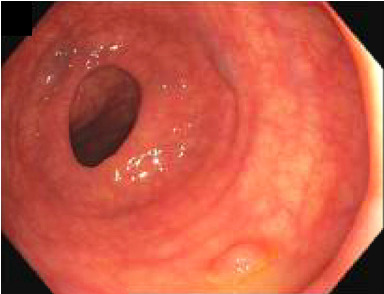
Colonoscopy image before biopsy showing a small, sessile (Paris classification 0–Is), smooth-surfaced polyp in the descending colon. The polyp is slightly elevated, lacks any visible ulceration or irregularities, and is set against healthy-looking surrounding mucosa.

A colonoscopy revealed five small polyps. Specifically, a single sessile polyp was found in the cecum, another in the transverse colon, one in the descending colon, two in the sigmoid colon, and one in the rectum (Figure 1). All polyps were completely removed using cold snare polypectomy and sent to the histopathology laboratory for analysis. In addition, evidence of diverticulosis was noted in the sigmoid colon.

**Figure 2:**
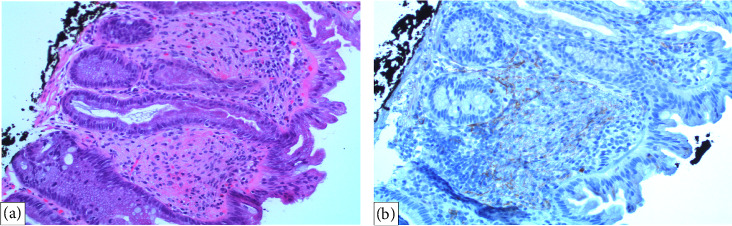
(a) A higher magnification view of bland spindle cell proliferation in the lamina propria associated with a sessile serrated lesion, stained with hematoxylin and eosin at 20x magnification. The surgical margins appear clear, suggesting complete excision. (b) Spindle cells exhibiting weak positivity for EMA, indicating at 20x magnification, supportive of the observed histological features.

Histopathological examination revealed that the four polyps located in the cecum, transverse colon, and rectosigmoid region were tubular adenomas with no signs of high-grade dysplasia. One polyp in the descending colon was identified as a mucosal perineurioma. IHC staining was positive for EMA but negative for smooth muscle actin (SMA) and SRY-related HMG-box 10 (SOX10) (Figure 2). Immunohistochemistry for GLUT-1 was performed; however, no residual lesion was identified on the slide. The patient was discharged from the gastroenterology clinic with a recommendation for a follow-up colonoscopy after 3 years.

### 3.2. Systematic Review

#### 3.2.1. Data Synthesis

**Figure 3:**
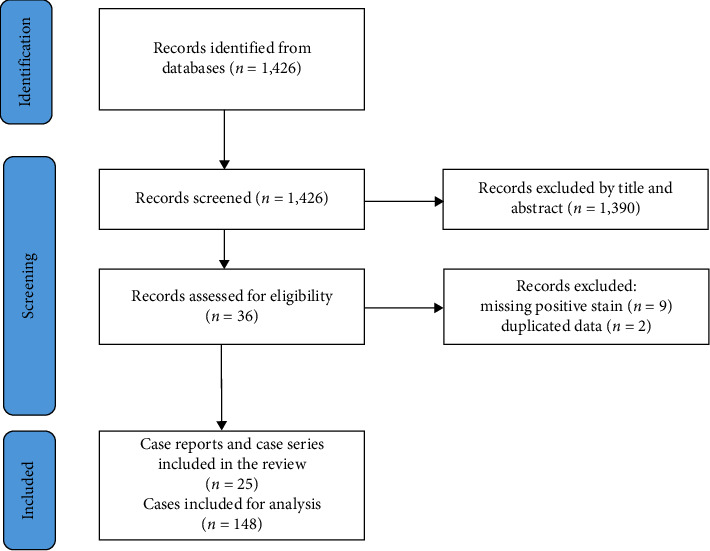
PRISMA flow diagram for the systematic review of perineuriomas and benign fibroblastic polyps in the GI tract.

The search strategy identified 1426 papers. After screening the titles and abstracts, 1390 papers were excluded. Full-text articles were then assessed for eligibility according to the abovementioned criteria, and 11 additional papers were excluded: nine due to missing immunohistochemistry stain positivity and two others because of data duplication (i.e., being included in a newer paper by the same author that incorporated data from the previously published work). The final results included 25 papers, comprising six case series and 19 case reports. Ultimately, 148 patients were included in the analysis (Figure 3 and Table 1).

### 3.3. GI Tract Perineuriomas

Table 1 presents descriptive statistics on perineuriomas from 25 case reports and case series. Most cases were published between 2014 and 2024. The age of the patients ranged from 24 to 87 years, with a mean of 51 years. The size of the polyps varied from 2 to 50 mm, with a mean of 4.18 mm. The majority of the patients were females (59.46%). The most common location was the sigmoid/rectosigmoid region (56.08%), followed by the rectum (14.76%) and transverse colon (6.08%). A total of 93.24% of the polyps were colonic, and 6.76% were noncolonic. Among the colonic polyps, 87.50% were located in the distal colon (left-sided), and 12.50% were located in the proximal colon (right-sided) (Table 2). The morphology was predominantly other/unspecified polyps (92.57%), with sessile polyps accounting for 6.08% and pedunculated polyps accounting for 1.37% of the polyps. Regarding the depth of invasion, 86.13% of the polyps involved the mucosa, while 13.87% invaded the submucosa. A high proportion of cases were positive for EMA (75%) and GLUT-1 (56.76%) and, to a lesser extent, for claudin-1 (44.59%) and collagen IV (47.30%) (Table 3). Symptomatic polyps accounted for 19.75%, while polyps identified through routine screening accounted for 80.24% of the cases.

### 3.4. Subgroup Analysis

#### 3.4.1. Colonic Polyp

**Figure 4:**
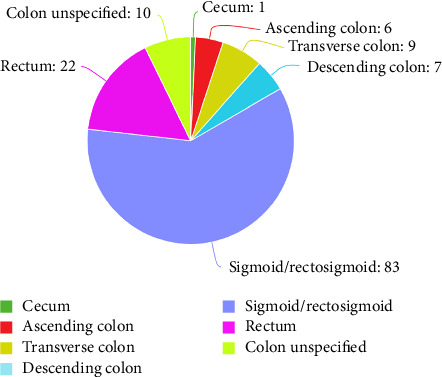
A pie chart showing the distribution of colonic polyps by region.

The results showed predominant involvement of the mucosal layer, comprising 90.63% of the patients. In contrast, the submucosa was less frequently involved, accounting for only 9.38% of the cases. The distribution of polyps varied significantly across different sections of the colon. The sigmoid/rectosigmoid region exhibited the highest percentage of involvement, at 60.14%. The rectum was also notably affected, representing 15.94% of the patients. The percentages in other regions, such as the transverse and descending colon, were lower, at 6.52% and 5.07%, respectively. The least involved region was the cecum, accounting for only 0.72% of the cases (Figure 4).

Regarding the laterality of findings, the left side of the colon was predominantly affected, with 87.5% of cases, compared to only 12.5% on the right side. Screening detected polyps in 85% of patients with right-sided colon involvement, and the same detection rate was observed in patients with left-sided colon involvement. The analysis of symptomatic versus screening-detected cases revealed comparable rates of symptomatic individuals, with 14.29% on the right side and 12.5% on the left side. The chi-square test yielded a value of (*χ*^2^ = 0.018) with a *p* value of 0.8933, indicating no statistically significant difference in the distribution of symptomatic and screening-detected cases between the two sides (Table 4).

#### 3.4.2. Characteristics of Polyps

Table 4 highlights key differences in colonic versus noncolonic perineurioma patients in terms of age, sex, polyp size, and detection methods. The ages of the patients with colon polyps ranged from 24 to 87 years, with an average age of 52.52 years, while those with noncolonic polyps ranged from 25 to 57 years, with an average age of 41 years (*p* < 0.001). Regarding sex distribution, females constituted 58.7% of the colonic cases and 70% of the noncolonic cases, while males accounted for 41.3% of the colonic cases and 30% of the noncolonic cases. The average size of the colonic polyps was 4.09 mm (SD = 3.79), which was significantly smaller than that of the noncolonic polyps (16.18 mm; SD = 14.2, *p* < 0.001). The detection methods also differed markedly: 87.30% of the colonic polyps were found during routine screening, while 11.11% of the noncolonic polyps were found during routine screening (*p* < 0.001). In addition, 88.89% of the noncolonic patients were symptomatic, while 12.6% of the colonic patients were symptomatic (*p* < 0.001). Compared with mucosal and submucosal perineuriomas, submucosal cases are more often symptomatic (66.67%), while mucosal cases are predominantly asymptomatic (90.7%), as detected mainly through screening. This difference was statistically significant, with a chi-square statistic of 17.371 (*p* < 0.001).

### 3.5. IHC Staining

In the subgroup analysis, which included 50 cases where three markers (EMA, GLUT-1, and claudin-1) were used in all samples, the results showed that EMA was positive in 84%, GLUT-1 in 94%, and claudin-1 in 88% of GI perineuriomas. Collagen IV was not included in the analysis because such staining was not performed in one of the studies. A chi-square test was used to compare the detection rates of the three markers. The chi-square statistic was 2.52, with a *p* value of 0.284. These findings indicate that there is no statistically significant difference in the expression of EMA, GLUT-1, or claudin-1 in GI perineuriomas.

### 3.6. Correlations Between Binary Variables and Clinical Features

**Figure 5:**
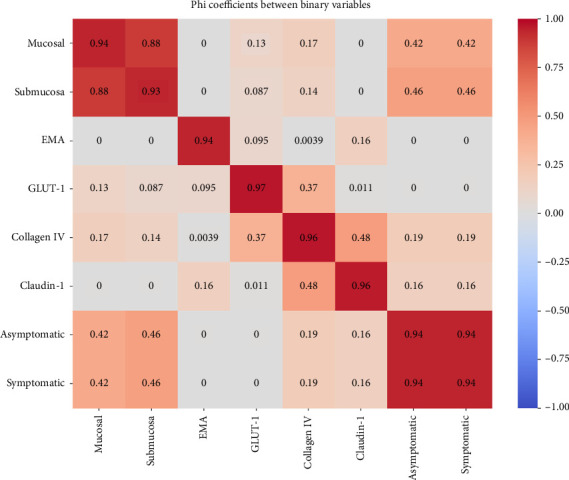
Heat map of phi coefficient values for associations among anatomical location, immunohistochemical staining, and clinical presentation.

Analysis of 68 cases revealed significant associations between anatomical location and symptomatology (*χ*^2^ = 14.76, *p*  < 0.001). Symptomatic cases demonstrated larger mean polyp sizes (12.77 mm) compared to asymptomatic cases (4.42 mm). Phi coefficient analysis revealed strong associations between specific IHC staining, particularly between EMA and GLUT-1 expression. Mucosal involvement showed a distinct pattern of distribution, with 44 cases showing mucosal involvement in asymptomatic patients compared to 5 cases in symptomatic patients, suggesting a potential prognostic indicator (Figure 5).

## 4. Discussion

Perineurioma is an extremely rare polyp that can manifest in various body locations [1, 2]. In the GI tract, information on perineuriomas has been reported primarily through case reports and case series rather than through large-scale epidemiological studies. Following the studies by Groisman and Polak-Charcon [7] and Zamecnik and Chlumska [42], who suggested reclassifying benign fibroblastic polyps with positive perineurial cell markers as perineuriomas, this study represents a comprehensive systematic review that integrates all related literature focusing on GI tract perineuriomas and benign fibroblastic polyps.

In this study, we summarized the findings of 148 cases with both perineurioma and benign fibroblastic polyps. The majority of the identified patients (59%) were female, with a mean age of 51 years. Our findings reaffirm that perineuriomas are most likely to be found in the colon, particularly the left-sided colon or distal to the splenic flexure, compared to other GI tract locations [38]. Noncolonic perineuriomas were identified in a small percentage (6.76%) of the cases evaluated in our study, with the majority of these polyps found in the stomach (4.73%). It appears that noncolonic perineuriomas differ from colonic perineuriomas in several aspects. First, the size in the noncolonic group was much larger than that in the colonic group, with a mean of 16.18 mm compared to 4.09 mm for the colonic group. In addition, most noncolonic perineuriomas are discovered following clinical manifestations rather than being found incidentally during routine endoscopy. However, it could be argued that routine colonoscopy in the targeted group increases the likelihood of finding these polyps incidentally in the colon.

When comparing mucosal and submucosal lesions, our findings align with previous suggestions [43] that submucosal lesions are more often associated with symptoms than mucosal lesions, with 66% of the submucosal polyps being symptomatic compared to only 18.92% of the mucosal polyps (*p* value < 0.001). According to the subgroup analysis for IHC, EMA positivity was 84% in 50 patients with perineuriomas. Given that EMA expression in the delicate cell processes of perineurioma cells can be limited and difficult to discern, we emphasize the previous recommendation by Groisman and Polak-Charcon [7] to perform at least two perineurial cell markers to achieve an accurate diagnosis and differentiate them from other spindle cell neoplasms.

The recurrence rates of perineuriomas have only been reported for non-GI tract cases. In a study by Hornick and Fletcher [44], recurrence was observed in just 2 out of 81 cases, with no signs of metastasis. These findings reinforce the benign nature of these tumors and suggest that recurrence is uncommon. However, no direct evidence exists regarding recurrence rates for GI tract perineuriomas, emphasizing the importance of documenting and studying such cases in the future.

The clinical implications of our findings highlight that perineuriomas in the GI tract are benign and generally do not require further management after removal. This reassurance is crucial for patients, alleviating concerns about the serious implications often associated with the term “polyp.” Educating patients about the benign nature of these lesions can enhance the quality of care by setting appropriate expectations and reducing unnecessary anxiety. For physicians, this knowledge supports efficient resource management, focusing on routine screenings and appropriate follow-up only when symptoms or new lesions are detected.

The strength of our systematic review lies in its comprehensive inclusion of all perineuriomas and colonic benign fibroblastic polyps throughout the entire GI tract, both upper and lower, using specific criteria for the application of perineurial cell markers, up to May 2024. Several limitations were observed in this study. Due to the rarity of these polyps, particularly in the GI tract, all data were extracted from case reports and case series, potentially affecting the generalizability of our findings. Another limitation is the limited amount of data, as these polyps are infrequent in the GI tract, leading us to rely solely on case reports and case series. In addition, our stringent inclusion criteria, especially regarding the IHC findings, excluded any report without evidence of perineurial marker positivity or any lesion with mixed histological features, which contributed to the limitations of our included studies. Last, due to the lack of follow-up reported in these reports, the recurrence rate of polyps is unknown or weakly estimated based on previous data. Moreover, the heterogeneous presentation and rarity of these lesions pose significant challenges in developing predictive models based on demographic and clinical predictors. However, we recognize the potential benefits of such tools and advocate for future collaborative efforts to collect more comprehensive data, which could lead to models that improve the early detection of perineuriomas.

Our recommendations include the following: We encourage physicians and pathologists to report uncommon locations of perineuriomas in the GI tract, as this will enhance the understanding and documentation of these cases. It is important to thoroughly describe the morphology of polyps when reporting any new cases, as detailed descriptions contribute to better diagnosis and classification. Although these polyps are benign, follow-up endoscopy might be considered to assess for recurrence.

## 5. Conclusion

In summary, perineuriomas or benign fibroblastic polyps in the GI tract are uncommon benign polyps that are predominantly found as mucosal polyps in the distal colon. Our systematic review of 148 cases revealed fewer, but more symptomatic, lesions in the stomach, small intestine, and esophagus. We recommend reporting uncommon polyp locations, detailing polyp morphology, and using at least two markers for classification.

## Figures and Tables

**Table 1:** Demographics and key features of the case reports and case series included in this review about GI perineuriomas.

ID	Authors, year (ref.)	No. of cases	Age or mean age	Gender	Location	IHC positivity
1	Vitale et al. (2024) [19]	1	52	Female	Noncolonic - Stomach	EMA GLUT-1
2	Abrari, Hasan, and Akhtar (2024) [25]	1	42	Female	Colonic - Rectum	EMA
3	Huber and DeRoche (2023) [26]	1	57	Female	Noncolonic - Stomach	EMA GLUT-1
4	Dias et al. (2023) [27]	1	51	Female	Colonic - Transverse colon	GLUT-1
5	Mesgun et al. (2022) [28]	1	57	Male	Colonic - Sigmoid/rectosigmoid	EMA
6	Tsuchiya et al. (2021) [29]	2	45	Female	Colonic - Transverse colon: 1 - Sigmoid/rectosigmoid: 1	EMA GLUT-1
7	Weaver et al. (2020) [30]	1	47	Female	Noncolonic - Stomach	GLUT-1
8	Kiryukhin et al. (2020) [31]	1	59	Female	Colonic - Sigmoid/rectosigmoid	EMA
9	Motta et al. (2018) [32]	1	40	Female	Colonic - Sigmoid/rectosigmoid	EMA Claudin-1
10	Otani et al. (2018) [33]	1	60	Female	Colonic - Sigmoid/rectosigmoid	EMA GLUT-1 Collagen IV
11	Jama et al. (2018) [34]	1	23	Male	Colonic - Sigmoid/rectosigmoid	EMA
12	Van Wyk, Van Zyl, and Rigby (2018) [16]	1	42	Male	Colonic - Transverse colon	EMA GLUT-1 Collagen IV
13	Hawes and Shi (2017) [35]	1	25	Female	Noncolonic - Stomach	GLUT-1
14	Matsui, Kashida, and Kudo (2016) [36]	1	51	Female	Noncolonic - Stomach	GLUT-1 Claudin-1
15	Fujino et al. (2014) [37]	1	51	Female	Colonic - Sigmoid/rectosigmoid	GLUT-1 Collagen IV
16	Groisman et al. (2013) [38]	60	60.75	Female: 30 Male: 30	Colonic - Cecum: 1 - Ascending colon: 4 - Transverse colon: 3 - Descending colon: 3 - Sigmoid/rectosigmoid: 35 - Rectum: 13	EMA GLUT-1 Collagen IV Claudin-1
17	Muguruma et al. (2012) [14]	1	45	Male	Noncolonic - Stomach	EMA Claudin-1
18	Wludarski et al. (2011) [20]	1	25	Male	Noncolonic - Small intestine	EMA Claudin-1
19	Pai et al. (2011) [39]	20	60.75	Female: 8 Male: 12	Colonic - Sigmoid/rectosigmoid: 7 - Rectum: 6 - Colon unspecified: 7	EMA
20	Agaimy et al. (2010) [40]	29	60.83	Female: 23 Male: 6	Colonic - Ascending colon: 2 - Transverse colon: 1 - Descending colon: 1 - Sigmoid/rectosigmoid: 22 - Rectum: 2 - Colon unspecified: 1	EMA GLUT-1 Collagen IV Claudin-1
21	Kelesidis et al. (2009) [18]	1	28	Male	Noncolonic - Esophagus	EMA GLUT-1 Collagen IV
22	Arrechea Irigoye et al. (2008) [41]	4	58.75	Female: 1 Male: 3	Colonic - Descending colon: 2 - Sigmoid/rectosigmoid: 2	EMA GLUT-1 Claudin-1
23	Zamecnik and Chlumska (2006) [42]	5	64.6	Female: 3 Male: 2	Colonic - Transverse colon: 1 - Sigmoid/rectosigmoid: 4	EMA GLUT-1 Claudin-1
24	Agaimy and Wuensch (2005) [15]	1	30	Female	Noncolonic - Stomach	EMA
25	Hornick and Fletcher (2005) [12]	10	49.36	Female: 8 Male: 2	Noncolonic - Small intestine: 1 Colonic - Transverse colon: 1 - Descending colon: 1 - Sigmoid/rectosigmoid: 6 - Colon unspecified: 1	EMA Claudin-1

**Table 2:** Descriptive statistics of polyps from case reports and case series.

**Variable**	**Range**	**Mean (±SD)**	

Age (*n* = 84)	24–87	51 (±14.87)	

Polyp size (mm) (*n* = 88)	2–50	4.18 (±3.69)	

**Variable**	**Category**	**Count**	**Percentage**

Sex (*n* = 148)	Female	88	59.46%
	Male	60	40.54%

Location (*n* = 148)	Esophagus	1	0.68%
	Stomach	7	4.73%
	Small intestine	2	1.35%
	Cecum	1	0.68%
	Ascending colon	6	4.05%
	Transverse colon	9	6.08%
	Descending colon	7	4.73%
	Sigmoid/rectosigmoid	83	56.08%
	Rectum	22	14.86%
	Colon unspecified	10	6.76%

Colonic and noncolonic (*n* = 148)	Colonic	138	93.24%
	Noncolonic	10	6.76%

Proximal colon and distal colon (*n* = 128)	Right-sided colon	16	12.5%
	Left-sided colon	112	87.50%

*Note: n* = cases with data used as the denominator for %.

Abbreviation: mm = millimeter.

**Table 3:** Morphology, invasion depth, and IHC staining.

Variable	Category	Count	Percentage (%)
Morphology (*n* = 148)	Other/unspecified polyp	137	92.57
	Sessile polyp	9	6.08
	Pedunculated polyp	2	1.37

Depth (*n* = 137)	Mucosa	118	86.13
	Submucosa	19	13.87

EMA positivity (*n* = 148)	Yes	111	75.00

GLUT-1 positivity (*n* = 148)	Yes	84	56.76

Collagen IV positivity (*n* = 148)	Yes	66	44.59

Claudin-1 positivity (*n* = 148)	Yes	70	47.30

*Note: n* = cases with data used as the denominator for %.

**Table 4:** Subgroup analysis of colonic versus noncolonic perineuriomas.

Variables	Colonic	Noncolonic	
Age range	24–87	25–57	
Age (±SD)	52.52 (±12.43)	41 (±12.53)	*p* value < 0.001
Sex (%) female	58.7%	70%	*p* value 0.495
Male	41.3%	30%	
Polyp range	2–20	4.5–50	
Polyp size (±SD)	4.09 (±3.79)	16.18 (±14.17)	*p* value < 0.001
Symptomatic (%)	12.6%	88.89%	*p* value < 0.001

*Note:* % = percentage.

Abbreviation: SD = standard deviation.

## Data Availability

The data that support the findings of this study are available from the corresponding author upon reasonable request.
